# Clinical aspects of common genetic Creutzfeldt-Jakob disease

**DOI:** 10.1007/s10654-012-9660-3

**Published:** 2012-03-02

**Authors:** Gabi Schelzke, Hans A. Kretzschmar, Inga Zerr

**Affiliations:** 1 Department of Neurology, Georg-August-University, 37075 Goettingen, Germany; 2 Department of Neuropathology, Ludwig-Maximilian University Munich, 81377 Munich, Germany

Creutzfeldt-Jakob disease (CJD) is a fatal rare neurodegenerative protein misfolding disease with a yearly incidence ranging from one to two per million. Most cases are of sporadic origin and the clinical phenotype, age at onset and disease duration are determined by the polymorphism at codon 129 of the prion protein gene (*PRNP*) and the prion protein type (PrP^Sc^) on western blot [1]. 10–15% of CJD cases are of genetic origin, caused by an autosomal dominant mutation in the *PRNP* at the short arm of chromosome 20 (genetic Creutzfeldt-Jakob disease—gCJD). So far, more than 30 mutations in the *PRNP* are known.

The worldwide most common mutations are E200K (A to G transition at codon 200 *PRNP* with substitution of lysine (K) for glutamate (E)) and V210I (G to A transition at codon 210 *PRNP* with substitution of isoleucin (I) for valine (V)) [2]. For the E200K mutation a cluster of cohorts were identified in Libyan Jews, Chile and Slovakia [3]. The clinical spectrum of these patients is reported similar to sCJD, except for the age at disease onset (average 10 years earlier disease onset in E200K) [2, 4]. The codon 129 *PRNP* genotype was identified to influence disease onset and duration (shorter mean disease duration in methionine-homozygous patients (3.7 ± 2.0 months) than in methionine/valine-heterozygous patients (7.84 ± 7.3 months)) [4]. About the V210I mutation several case reports were published, but actually no clear comprehensive clinical picture exists. In general, the mean age at onset ranged at 50 years, the mean disease duration at 4 months in patients with the V210I mutation [2, 5]. Comprehensive data from the literature about both mutations suggest that the majority of cases feature a similar phenotype like sporadic CJD in absence of a positive family history of prion diseases. In our study, we analyzed the clinical phenotype and results of diagnostic tests in patients with gCJD E200K and V210I and compared them to sCJD control groups matched by age, gender and *PRNP* 129 codon genotype (for both mutations separate sCJD control groups in the same patients number). We then compared the results between the mutations and the control group. By means of statistical relevance the PrP type was not considered in this study, as it was not available in most cases. All patient data analyzed in this study derived from the German CJD surveillance unit since it was established in 1993. The E200K group comprised 23 patients with a median age at onset of 63 years (range 29–75 years) and median disease duration of 9 months (range 2–19 months), thereof 15 female and 8 male patients (Table 1). In 47% of these patients at least one more E200K mutation affected relative was identified. Twelve patients were homozygous for methionine, eleven heterozygous for methionine/valine, whereas in eight of the heterozygous patients the mutation was linked to the methionine allele (E200K–M) and in three patients it was linked to the valine allele (E200K–V). The mean age at disease onset did not differ between homozygous and heterozygous patients (MM 65 ± 10 years, MV 62 ± 12 years, *p* = 0.728), but disease duration varied significantly (MM 6 ± 3 months, MV 12 ± 5 months, *p* ≤ 0.001). When compared to sCJD, no significant differences in those parameters were detected. There was a significant difference between the E200K–M (65 years) and E200K–V (61 years) connected mutation carrier in age at onset (*p* = 0.025), however no difference in disease duration (E200K–M 6.5 months, E200K–V 11.3 months; *p* = 0.808). The E200K–V patients were significantly younger at disease onset than the heterozygous sCJD patients (E200K–V 51 years, MV sCJD 64 years; *p* = 0.036), whereby no differences between E200K–M and methionine homozygous sCJD patients were detectable. No divergence of age at onset and disease duration existed between cases with a positive family history and cases without a positive family history of the E200K mutation. The V210I cohort of 16 patients (MM = 13, MV = 3, all linked to methionine), thereof 9 female and 7 male without positive family history of prion diseases in any case (Table 1). The mean age at onset was 63 years (range 45–80 years), the mean disease duration 6 months (range 1–20 months). Homozygous and heterozygous patients had a similar average age at onset (MM 61 ± 11 years, MV 55 ± 8 years, *p* = 0.238) and disease duration (MM 5 ± 5 months, MV 2 ± 0.4 months, *p* = 0.181). Frequent clinical symptoms in both mutations at disease onset were part of a prodromal phase (vertigo, personality change and headache) followed by dementia and ataxia. All sensory disturbances as well as half of all psychiatric features in both mutations appeared at disease onset and during the disease all typical symptoms of CJD were presented (Table 2). The mutation carriers presented a variety of clinical symptoms similiar to the sCJD control group. By analysing symptom appearance (onset) we could not verify any differences between the E200K patients and the tightly matched sCJD control group. However, between homozygous and heterozygous E200K patients myoclonic jerks (*p* = 0.007), pyramidal signs (*p* = 0.015), extrapyramidal symptoms (*p* = 0.003) and psychiatric features (*p* = 0.003) appeared significantly later in disease course in heterozygous patients. Compared to V210I psychiatric features occured later in sCJD (*p* = 0.008), homozygous V210I mutation carrier developed cerebellar signs later than heterozygous patients (*p* = 0.019). The E200K and V210I mutation distinguished by the fact that dementia (*p* = 0.019), pyramidal signs (*p* = 0.041), extrapyramidal signs (*p* = 0.004) and psychiatric features (*p* < 0.001) appeared later in the E200K mutation, whereby both groups were not matched by age, gender or *PRNP* polymorphism. The results of the diagnosing tests revealed a positive CSF 14-3-3 protein in 91% of the E200K mutation carrier (MM = 100%, MV = 82%) and in 100% of the V210I mutation carrier. The median level of tau protein in E200K mutation ranged at 6400 pg/ml (range 1300–24638 pg/ml). Codon 129 *PRNP* homozygous mutation carrier had a trend towards lower tau levels than heterozygous patients (MM = 8628 pg/ml; MV = 4172 pg/ml; *p* = 0.071). Tau values differed substantially in homozygous and heterozygous sCJD patients (MM = 11472; MV = 2319 pg/ml; *p* = 0.004). In the E200K mutation the tau and the age at onset presented with an inverse correlation whereby we observed lower tau values in older patients (r = −0.555, *p* = 0.007). In the V210I mutation the median tau protein levels ranged at 7640 pg/ml (MM = 7628 pg/ml, MV = 7686 pg/ml, *p* = 0.991) with no differences between *PRNP* codon 129 homozygous and heterozygous patients. In sCJD controls a significant difference between homozygous and heterozygous patients was similarly presented (MM = 8762 pg/ml, MV = 1529 pg/ml; *p* ≤ 0.001). The sCJD control group and patients with gCJD presented no differences between frequency of positive CSF 14-3-3 protein or values of tau protein. 27% of the E200K patients and 44% of the V210I mutation carrier demonstrated typical periodic sharp wave complexes (PSWC) on EEG. On MRI, 89% of the E200K mutation carrier and 95% of the sCJD control patients met the current MRI criteria regarding to hyperintensities in basal ganglia and/or cortical areas (Table 2). In 60% of the E200K mutation carrier we found white matter lesions (WML), whereby patients with WML were significantly older in contrast to the patients without WML (median age WML = 63 years; median age no WML = 58 years; *p* ≤ 0.05). In the sCJD control group the frequency of WML was even higher with presence in 90%. We did not detect a statistically significant difference in the age of patients with WML as compared to those without WML. In V210I mutation all of the patients presented with a positive MRI scan for CJD (sCJD 83%), we also found WML (30%), but less frequently than in the E200K mutation. Statistical analyses revealed no variances between the mutations and sCJD control groups in results of EEG and MRI.

**Table 1:** Demographic data of the E200K and V210I *PRNP* mutation carrier

Demographic data	E200K			V210I		
	Total n = 23	MM n = 12	MV n = 11	Total n = 16	MM n = 13	MV n = 3
Male:female	1:1.8	1:1	4.5:1	1:1	1.6:1	1:2
Age at onset (years)	63 (29–75)	65 (43–73)	62 (29–75)	63 (45–80)	61 (52–80)	55 (48–64)
Disease duration (months)	9 (2–19)	6 (2–11)	12 (5–19)	6 (1–20)	5 (1–20)	2 (2–3)

**Table 2:** Results of the diagnosing tests and frequency of clinical symptoms in E200K mutation carrier and V210I mutation carrier as well as the sCJD control groups (different sCJD controls matched by age, gender and codon 129 *PRNP* genotype separately for both mutations with the same number of patients)

Diagnostic tests	E200K	sCJD	V210I	sCJD
14-3-3				
MM (%)	100	100	100	100
MV (%)	82	82	100	67
Median tau [pg/ml]				
MM	8628	11472	7628	10716
MV	4172	2319	7686	2348
EEG				
MM (%)	27	42	46	50
MV (%)	27	10	33	50
MRI				
MM (%)	88	90	100	80
MV (%)	90	100	100	100

Clinical signs (%)	Disease onset		During disease		Disease onset		During disease	
	E200K	sCJD	E200K	sCJD	V210I	sCJD	V210I	sCJD
Prodromal phase	74	83	74	83	75	81	75	81
Dementia	61	48	87	87	69	69	88	100
Cerebellar signs	52	78	100	100	63	75	100	100
Myoclonic jerks	9	4	65	87	25	19	88	88
Pyramidal signs	35	17	83	57	13	31	38	75
Extrapyramidal signs	13	9	70	52	31	6	81	75
Psychiatric features	30	48	70	70	13	25	31	63
Sensory disturbances	30	13	30	13	19	6	38	6

In conclusion, we demonstrated that patients with the E200K and the V210I mutation exhibited a similar clinical syndrome as sporadic CJD patients and presented similar findings in the diagnosing tests, given that they were matched to age, gender and codon 129 *PRNP* polymorphism. In contrast to the V210I mutation carrier, the E200K mutation clinical symptoms of dementia, pyramidal signs, extrapyramidal signs and psychiatric features appeared later in disease course what could be a distinctive feature. An aspect of our patients was that we did not find strategic differences between *PRNP* codon 129 polymorphism in each group, whereby this parameter represents one of the strongest susceptibility factor of phenotypic variability in sCJD. Another interesting result was the detection of a high rate of WML, whereby the reasons for white matter involvement in CJD have not been well explained yet. Summarizing, in absence of a positive family history and no obvious indications to distinguish the origin of the CJD, only wide genetic testing of all CJD patients allows an accurate diagnosis of a genetic disorder.
